# From evasion to elicitation: A fungal effector turned into defense trigger

**DOI:** 10.1093/plphys/kiaf582

**Published:** 2025-11-12

**Authors:** Marcella Teixeira

**Affiliations:** Assistant Features Editor, Plant Physiology, American Society of Plant Biologists; Department of Plant Pathology, Washington State University, Pullman, WA 99163, USA

To succeed in parasitism, plant pathogens deploy an arsenal of secreted proteins known as effectors, which can reshape plant physiology to their advantage, modulating immune responses or even reprogramming plant cell fate (Hogenhout et al. 2009). They are often grouped by conserved motifs, such as the LysM domain, found in different fungi (Marshall et al. 2011; Kombrink and Thomma 2013). Plants also encode LysM-domain proteins like OsCEBip in rice, which perceives chitin fragments from fungal cell walls and triggers defense signaling through dimerization with its co-receptor OsCERK1 (Tanaka et al. 2013; Albert et al. 2020). To avoid this surveillance, fungi use LysM effectors to sequester these chitin fragments, hiding from the plant receptors (de Jonge et al. 2010).

In this issue of *Plant Physiology*, Peng et al. (2025) uncover an example of a molecular arms race involving *Lasiodiplodia theobromae*, a grapevine pathogen notorious for its wide host range and extensive secretome of over 900 proteins (Yan et al. 2018). Among these, the authors identified 6 LysM proteins, including LtLysM2, which contains a secretion signal and 2 LysM domains. Using yeast secretion trap and affinity precipitation assays, they confirmed LtLysM2's secretion and chitin-binding ability, suggesting that *L. theobromae* uses this effector to evade a yet undescribed grapevine chitin receptor (Fig.).

**Figure.**
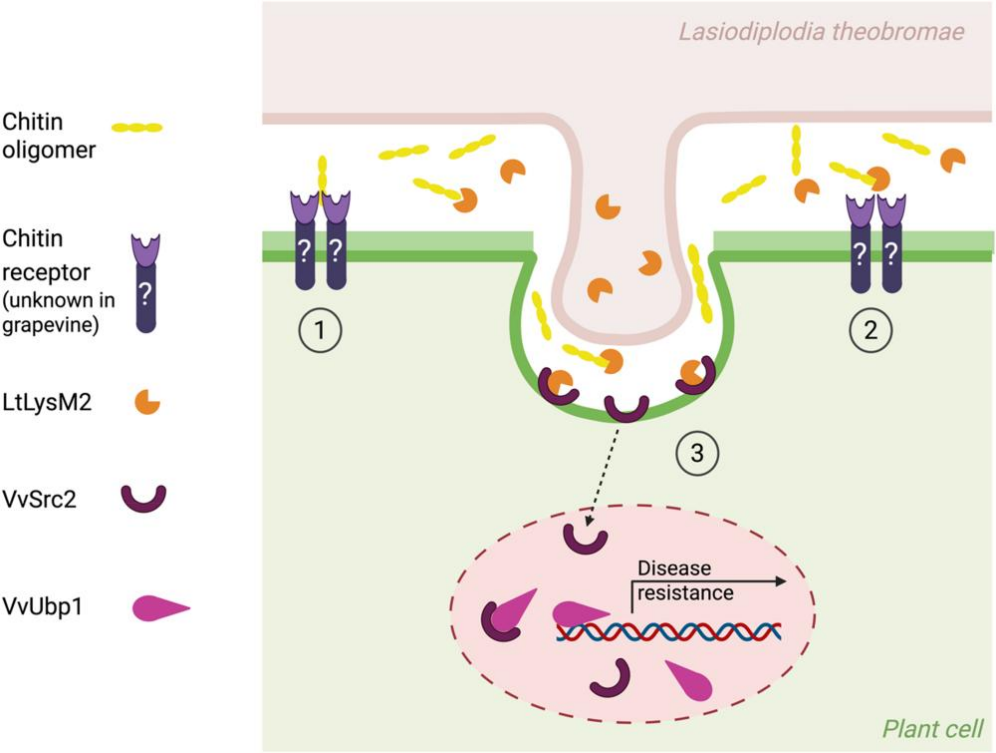
Turning stealth into signal: grapevine detects a fungal effector to regain immunity. 1. Plant receptors can detect fungal chitin during infection, which results in chitin-triggered immunity. 2. To prevent this detection, *L. theobromae* secretes LtLysM2, which binds to chitin and suppresses chitin-mediated detection. 3. VvSrc2, a membrane-localized protein from grapevine, interacts with LtLysM2, resulting in inhibition of the immunity-suppressive function of LtLysM2, possibly by competition for chitin binding and occupancy effect. This interaction promotes VvSrc2 nuclear accumulation, where VvSrc2 associates with the nuclear-localized protein VvUbp1. Overexpression of *VvSrc2* contributes to VvUbp1 protein accumulation and programmed cell death. VvUbp1, a putative RNA-binding protein homologous to the *Triticum aestivum* TaUBA2C, possibly activates host immunity through recruiting the pre-mRNA of ROS burst-related genes and mediating their expression. (Adapted from Figure 7, Peng et al. 2025; Created in BioRender. Teixeira, M. (2025) https://BioRender.com/uhe7oql).

When grapevine (*Vitis vinifera*) shoots were inoculated with *L. theobromae* overexpressing *LtLysM2*, the resulting lesions were longer than with the control fungus, whereas silencing the gene reduced symptoms, establishing its role in virulence. A yeast 2-hybrid (Y2H) screen revealed that LtLysM2 interacts with VvSrc2, a grapevine protein with a C2 domain, transmembrane domain, and nuclear localization signal. C2 domains, which often bind Ca^2+^, are known players in plant defense signaling and have been shown to be involved in different processes, including regulation of programmed cell death (Yang et al. 2007) and response to abiotic stresses (de Silva et al. 2011).

The interaction between LtLysM2 and VvSrc2 was confirmed by Y2H, pull down assay, and bimolecular fluorescence complementation assay. Transient expression of VvSrc2 in *N. benthamiana* confirmed its accumulation in the cell membrane and nucleus. Investigation of truncated fragments using transient expression showed that the C2 domain and the nuclear localization signal motif were associated with membrane and nuclear localizations, respectively. Co-infiltration of *N. benthamiana* with LtLysM2 and VvSrc2 resulted in increased protein amount and stronger nuclear localization of VvSrc2, revealing an effect of LtLysM2 on VvSrc2 accumulation and localization.

Since LtLysM2 binds chitin, it could suppress chitin-triggered reactive oxygen species (ROS) production. The authors showed that LtLysM2 suppressed the chitin-mediated ROS burst in *N. benthamiana* and VvSrc2 inhibited this suppression, implying that VvSrc2 interacts with LtLysM2 to disrupt its function in disease progression. To investigate the role of VvSrc2 in defense regulation, Peng et al. (2025) generated *N. benthamiana* transgenic lines overexpressing VvSrc2. Inoculation of these transgenic lines with wild-type fungus showed that VvSrc2 suppressed disease progression. These results show that by detecting LtLysM2, grapevine has a way to counterattack the attempt by *L. theobromae* to prevent chitin-mediated defense responses (Fig.).

Transcriptome analysis of the *N. benthamiana* transgenic lines overexpressing VvSrc2 revealed differential expression of genes related to defense responses and ROS, which were validated using reverse transcription quantitative PCR. Additionally, 3,3-diaminobenzidine staining confirmed a higher level of ROS accumulation in the transgenic lines upon inoculation with *L. theobromae*, suggesting that VvSrc2 acts as a positive regulator of defense responses.

To understand how VvSrc2 enhances defense, the authors further investigated its nuclear accumulation using Y2H to search for plant interactors. Among the candidates, they identified VvUbp1, an RNA-binding protein homologous to a wheat immunity regulator that promotes ROS production and cell death (Li et al. 2022). The interaction between VvSrc2 and VvUbp1 was confirmed using Y2H, bimolecular fluorescence complementation, and in vitro pull-down assay. Transient expression in *N. benthamiana* confirmed VvUbp1 accumulation in the nucleus and showed its expression leads to cell death. Evaluation of ROS burst-related genes revealed that VvUbp1 infiltration results in increased expression of these genes, suggesting that VvUbp1 mediates cell death by activating ROS burst related genes. Interestingly, co-infiltration of VvUbp1 and VvSrc2 results in stronger cell death, implying that VvSrc2 helps VvUbp1 mediated-ROS burst. This illustrates how grapevine uses LtLysM2 to mount a counterattack, detecting the effector itself to trigger stronger immunity (Fig.).

Together, these findings show a sophisticated molecular dialogue between *L. theobromae* and grapevine (Fig.). In this interaction, the fungus secretes the LysM effector LtLysM2 to capture chitin fragments and suppress immune recognition. Grapevine, however, has evolved VvSrc2, a C2-domain protein that detects LtLysM2 and recruits the RNA-binding protein VvUbp1 (Fig. 1). This interaction restores defense signaling, leading to ROS accumulation and activation of disease resistance genes. The work by Peng et al. (2025) illustrates how plants can turn a pathogen's stealth strategy into a trigger for their own protection, showcasing the dynamic coevolution that defines plant-microbe interactions.

## Data Availability

No data is generated in this study.
